# Evaluation of oxidative stress effects on different macromolecules in adult growth hormone deficiency

**DOI:** 10.1371/journal.pone.0236357

**Published:** 2020-07-20

**Authors:** Antonio Mancini, Carmine Bruno, Edoardo Vergani, Francesco Guidi, Flavia Angelini, Elisabetta Meucci, Andrea Silvestrini

**Affiliations:** 1 Dipartimento di Medicina e Chirurgia Traslazionale, Università Cattolica del Sacro Cuore, Rome, Italy; 2 Fondazione Policlinico Universitario A, Gemelli IRCCS, Rome, Italy; 3 Dipartimento di Scienze biotecnologiche di base, cliniche intensivologiche e peri-operatorie, Università Cattolica del Sacro Cuore, Rome, Italy; University of Arkansas for Medical Sciences College of Pharmacy, UNITED STATES

## Abstract

Adult growth hormone deficiency (GHD) is being increasingly recognized to cause premature mortality exacerbated by oxidative stress. A case-control observational study has been performed with the primary objective of evaluating new parameters of oxidative stress and macromolecular damage in adult GHD subjects: serum nitrotryptophan; Total Antioxidant Capacity expressed as LAG time; urinary hexanoil-lysine; urinary dityrosine and urinary 8-OH-deoxyguanosine. GHD was diagnosed using Growth Hormone-Releasing Hormone 50μg iv+arginine 0,5 g/Kg test, with a peak GH response <9 μg /L when BMI was <30 kg/m^2^ or <4 μg/L when BMI was >30 kg/m^2^. Patients affected by adult GHD were divided into three groups, total GHD (n = 26), partial GHD (n = 25), and controls (n = 29). Total Antioxidant Capacity, metabolic and hormonal parameters have been determined in separate plasma samples; nitrotryptophan in serum samples; hexanoil-lysine, dityrosine, 8-OH-deoxyguanosine in urine samples. Assessment of hexanoil-lysine exhibited a trend to increase in comparing total GHD vs partial and controls, although not significant. Values of 8-OH-deoxyguanosine did not significantly differ among the three groups. Significant lower levels of dityrosine in partial GHD vs total and controls were found. No significant difference in nitrotriptophan serum levels was found, while significantly greater values of Total Antioxidant Capacity were showed in total and partial GHD vs controls. Thus, our result confirm that oxidative stress is increased both in partial and total adult GHD. The lack of compensation by antioxidants in total GHD may be connected to the complications associated to this rare disorder.

## Introduction

Adult growth hormone deficiency (GHD) is defined as a clinical expression of a reduced GH secretion caused by congenital or acquired diseases affecting the hypothalamus or pituitary gland. Adults who develop GHD may report central weight gain, difficulty to lose weight, dry skin, fatigue and reduced quality of life [1].

Interestingly, experimental evidences, both in childhood and adulthood, have associated the GHD condition to oxidative stress (OS) [2]. OS could be defined as the imbalance between the production of oxidants and antioxidants in favour of the former, leading to a disruption of redox signalling and/or molecular damage [3]. However, discordant data concern growth hormone (GH) effects on antioxidant levels are reported. GH, for instance, influences the function of immune cells, inhibiting apoptosis in neutrophils by downregulation *Fas* expression and increasing reactive oxygen species (ROS) production [4]. Furthermore, GH increases ROS production in monocytes, even though no data on apoptosis in these cells have been reported [5]. Moreover, results in experimental animal models are contradictory. In Wister rats on caloric restriction GH/insulin treatment decreases ROS production and DNA damage in heart mitochondria, whether in the liver the same treatment decreases ROS production, but enhances DNA damage, thus suggesting pleiotropic effects of GH depending on involved tissue [6]. Mice with knock-out for the receptor of Growth Hormone (GHR-KO) have increased antioxidant defences with different total antioxidant capacity (TAC) according to sex [7]. In Ames dwarf mice with congenital TSH, prolactin and GH deficiency the enzymatic antioxidants appear to be increased even though scavengers, on the contrary, are decreased [8]. In human being with pre-pubertal GHD, GH treatment seems to increase antioxidant capacity [9]. Moreover, Scacchi et al. show increased lipid peroxidation in adult GHD, counteracted by GH treatment [10]. Additionally, some papers report the relationship between OS and insulin resistance in GHD patients [11,12]. In adults, GHD, indeed, shares with metabolic syndrome clinical and biochemical features even though differential pattern of antioxidant response and inflammatory mediators may be involved [13,14].

OS can be related to proteins, nucleic acids and lipids damage [15], in turn associated to an augmented cardiovascular and oncological risk [16,17]. Confirming, adult GHD, a condition of increased OS, is characterized by hypercoagulability and faster atherosclerosis development [18] and increased incidence of cancer [19]. Also partial GHD in adulthood, defined as a subnormal GH response to stimulation tests not so severe to require a replacement therapy, has been associated to altered cardiovascular and metabolic features [20–23].

Since the early identification of oxidative molecular damage may represent a possible strategy to contrast the appearance of the previous mentioned complications, we aimed to extend our findings with a specific focus on parameters of oxidative stress, not previously evaluated in adult GHD subjects, which assess damages on macromolecules (e.g. lipids, proteins, nucleic acids). Thus, in this case-control observational study, the primary objective was the evaluation of new parameters of oxidative stress in adult GHD. Specifically, we measured serum nitrotryptophan (N-Try), as a biomarker of protein nitration, urinary hexanoil-lysine (HEL) as parameter of lipidic oxidation; urinary dityrosine (DT), as a parameter of protein oxidation, urinary 8-OH-deoxyguanosine (8-OHdG) as a parameter of DNA oxidation, and the evaluation of Total Antioxidant Capacity (TAC), while secondary objectives were the definitions of metabolic and lipid pictures in this clinical background.

## Materials and methods

### Study population

The patients involved in this study consisted of 80 subjects (36 males, 44 females), admitted to the University Hospital “Policlinico Gemelli” Dept. of Internal Medicine, and enrolled (from 2016 to 2018) after being given an explanation of purposes and nature of the study, conducted in accordance with the declaration of Helsinki, as revised in 2013. The study protocol was approved by “Fondazione Policlinico Gemelli” ethical committee (Protocol sf 39612/16).

After being given a written consent, GHD untreated patients and controls of both sexes were included in the study.

Exclusion criteria were: age under 18 or over 70; obesity of genetic origin or related to other endocrine diseases; history of cranial hypertension or active cranial hypertension, decompensated type 1 or 2 diabetes mellitus; autoimmune diseases under immunosuppressive treatment; corticosteroid treatment (except for topic, inhalatory and oral hydrocortisone as replacement regimen); other diseases characterized with low insulin-like growth factor-1 (IGF-1) such as liver disease, malabsorption and malnutrition; active malignancy. No patients received GH therapy at the study enrollment.

GHD was diagnosed with dynamic test, using Growth Hormone-Releasing Hormone (GHRH 50 μg intravenous + arginine 0,5 g/Kg) (GHRH/ARG).

26 patients, 13 males and 13 females, presented a peak GH response < 9 μg/L (or <4 μg/L when BMI ≥ 30 kg/m^2^) and were classified as total GHD group; 25 patients, 12 males and 13 females, with GH peak between 9 and 16 μg/L were considered as partial GHD group. Finally, 29 subjects, 11 males and 18 females, matched for age and sex, with GH peak > 16 μg/L were included as control group.

Within the total GHD group, different etiologies were found: 10 cases of empty sella syndrome, 11 cases of idiopathic isolated GHD, 1 case of Arnold-Chiari syndrome, 1 case of Sheehan’s syndrome, 1 case of pituitary cist, 1 case of post-surgical hypopituitarism, 1 case of pituitary infarction.

Patients underwent urine and venous sampling at 8 a.m, after an overnight fasting, collecting blood samples by two pyrogen-free tubes, one containing heparin as anticoagulant, and one without anticoagulant to obtain serum.

### Serum evaluation for hormones and N-Try

The sample collected into vacutainer tube(s) containing no anticoagulant was incubated in an upright position at room temperature for 30–45 min (no longer than 60 min) to allow clotting and then centrifuged at 2500 rpm for 15’ at 4°C without brake. Supernatant (serum) was collected and stored at -80°C until use.

The following hormonal parameters were determined: freeT3 (fT3), freeT4 (fT4), thyroid-stimulating hormone (TSH) and adrenocorticotropic hormone (ACTH). The methods used in hormone assays were: electro-chemo-luminescent immunoassay for ACTH (normal range 10–55 pg/ml); chemo-luminescent immunoassay for insulin (n.r. 3–20 μUI/ml) TSH (n.r. 0.35–2.80 μUI/ml), fT3 (n.r. 2.4–4.2 ng/ml), fT4 (n.r. 8.5–16.5 pg/ml), IGF-1 (n.r. 80–330 ng/ml). Serum concentrations of IGF-1 were measured by using immunochemiluminometric assays on a Roche Modular E170 analyzer (Roche Diagnostics, Indianapolis, IN, USA). The intra- and inter-assay CV for all hormones were, respectively, < 5.0% and < 7.0%.

Western Blot analysis was applied to quantify N-Try (formed by peroxynitrite and tryptophan) as biomarker of protein nitration; nitration of tryptophan residues is an oxidative modification of proteins connected with cellular signaling, vasorelaxation and, possibly, carcinogenic damage [24]; before the analysis albumin was removed from serum samples following the protocol reported by Chen et al [25]. Briefly, 100 μl of serum was precipitated by addition of four volumes of ice-cold acetone containing 10% w/v TCA and incubated at -20°C for 90 min. The mixture was centrifuged at 15000 g for 20 min at +4°C and the pellet washed with an addition of 1ml of ice-cold acetone followed a centrifugation at 15000 g for 20 min at +4°C. The pellet was re-suspended in 100 μl of a buffer containing 25 mM HEPES, pH 7.4, 0.1% SDS, 4 M urea; the mixture was hated at 55°C for 10 min to permit a complete solubilisation. After albumin removal, 15 μl of sample plus 5 μl of loading buffer was loaded into a 10% acrylamide/bis-acrylamide gel. After running and transfer blot, the nitrocellulose was blocked for 1 hour at RT with 5% non-fat milk and then incubated with an anti-Nitrotryptophan monoclonal antibody (Japan Institute for the Control of Aging (JaICA), Nikken SEIL Co., Ltd.) (100ug/ml) diluted 1/500 or with anti-Transferrin antibody (0,7mg/ml) (ad109503, Abcam plc, USA) diluted 1/5000. As a secondary antibody, we used a biotinylated anti-mouse 1/1000 (ab97023, Abcam plc, USA) for nitro-tryptophan or an anti-rabbit (ab97051, Abcam plc, USA) 1/15000 for transferrin. Luminol reagent (XLS025.0000, Cynagen srl., Bologna, Italy) was added and quantified with Chemidoc instrument and ImageLab software (Bio-Rad California 94547, USA). Data were expressed as fold increase (protein investigated/protein positive control—nitro-tryptophan/transferrin).

### Plasmatic evaluations

The sample collected into pyrogen-free tubes containing heparin was centrifuged within one hour at 4000 rpm for 15 minutes; separate plasma aliquots were stored at -80°C until assayed. Assessment of biochemical and metabolic parameters was performed, including plasmatic glucose, total cholesterol, low-density lipoprotein (LDL), high-density lipoprotein (HDL), triglycerides and uric acid. Plasma concentrations of glucose, total cholesterol, HDL-cholesterol, triglycerides, uric acid were measured using enzymatic assays by an Olympus AU2700 chemistry analyzer (Olympus America Inc., Center Valley, PA, USA). The intra-and inter-assay coefficients of variation (CV) for total cholesterol and triglycerides were < 1.5%, and < 2.5%, respectively. The intra-and inter-assay CV for HDL-cholesterol were < 2.5%, and < 3.0%, respectively. LDL cholesterol was calculated by Friedewald’s equation: LDL = total cholesterol—(HDL + triglycerides/5). HOMA index was calculated according to the formula {[fasting insulin (μU/ml)] * [fasting glucose (mmol/l)]}/22.5 [26].

TAC was evaluated by spectrophotometric method, with a modification of the method developed by Rice-Evans and Miller [27], as previously described [28]. The method is based on the antioxidants inhibition of the absorbance of the radical action 2,2I-azinobis (3-ethylbenzothiazoline-6 sulphonate) (ABTS^.+^) formed by interaction between ABTS (150 μM) and ferrylmyoglobin radical species, generated by activation of metamyoglobin (2.5 μM) with H_2_O_2_ (75 μM). Aliquots of the frozen plasma were thawed at room temperature and 10 μl of the samples were tested immediately. The manual procedure was used with only minor modifications, i.e., temperature at 37° C to be in more physiological conditions and each sample assayed alone to carefully control timing and temperature. The reaction was started directly in cuvette through H_2_O_2_ addition after 1 min equilibration of all other reagents (temperature control by a thermocouple probe, model 1408 K thermocouple, Digitron Instrumentation Ltd, Scunthorpe, United Kingdom) and followed for 10 min under continuous stirring, monitoring at 734 nm, typical of the spectroscopically detectable ABTS^.+^. The presence of chain-breaking antioxidants induces a lag time (the Lag phase) in the accumulation of ABTS^.+^ whose duration is proportional to the concentration of this type of antioxidants. Antioxidant capacity afforded by chain-breaking antioxidants is expressed as length of Lag phase (LAG, sec). Trolox, a water-soluble tocopherol analog, was used as a reference standard and assayed in all experiments to control the system. Absorbance was measured with an Agilent 8453 UV/Vis spectrophotometer (Palo Alto, CA, USA) equipped with a cuvette stirring apparatus and a constant temperature cell holder. Measurements of pH were made with a PHM84 Research pH meter (Radiometer, Copenhagen, Denmark); the electrode response was corrected for temperature. Unless otherwise stated, experiments were repeated two to three times; qualitatively similar results were obtained with individual values varying < 8%. In the Lag mode, the assay mainly measures non-proteic and non-enzymatic antioxidants that are primarily extracellular chainbreaking antioxidants, such as ascorbate, urate and glutathione.

### Urinary evaluations

After collected urine was immediately aliquoted and stored at − 80 °C. Before assayed, urine was centrifuged at 1500 g at 4 °C for 15 min and the supernatant was used for the measurement. In urine we evaluated the additional indexes of oxidative stress:

- HEL adduct, as biomarker of lipid peroxidation, by ELISA method; it is a novel lipid hydroperoxide-modified lysine residue that is formed by oxidative modification by oxidized omega-6 fatty acid such as linoleic acid or arachidonic acid. HEL is therefore a biomarker for initial stage of lipid peroxidation [29].
- Urinary DT, as biomarker of protein oxidation, by ELISA method; DT is a tyrosine dimer derived from tyrosyl radicals formed by ROS, metal-catalyzed oxidation and peroxidases [30].
- Urinary 8-OHdG, by ELISA method; it is an oxidized nucleoside of DNA; upon DNA repair, it is excreted in urine and is the most frequently detected parameter of DNA lesions [31].

For HEL and DT measurements, the sample was diluted in 3 parts of saline buffer (0.9% NaCl in distilled water) as declared in the datasheet of each kit. All the analyzes have been performed following the manufacturer's instructions. All ELISA kits have been purchased from JaICA, Japan.

### Statistical analysis

The statistical analysis was performed using Stata 13 software. The planned sample size of minimum 25 cases for each group was not based on formal power estimates, due to the lack of any information on the size of the minimal difference worth detecting for each of the study parameters. Instead, the sample size was established based on considerations of statistical practicality (with 30+30 subjects a normal distribution of the mean can be usually assumed) and feasibility, due to the rarity of GHD disease. With this sample size, the study has power >80% to detect any difference between cases and controls that is as large or greater than 75% of the standard deviation of that specific parameter. Considering that the comparison concerns differences between diseased subjects and healthy controls in physiologic parameters of oxidative stress, smaller differences are of little, if any, interest.

According to Kolmogorov-Smirnoff test, variables followed standard distribution. The statistical analysis was carried out using the variance analysis (F test—ANOVA) to study the differences among groups and, when significant, the Student T test for unpaired data, in two groups comparison. Moreover, linear regression analysis was performed, with the calculation of the Pearson coefficient. Linear correlation analyses were executed to evaluate the correlation between variables. The level of significance has been set at 0.05.

Finally, Pearson’s coefficient was used to investigate the correlation between each parameter of oxidative stress (i.e. LAG, HEL, DT, 8-OH-dG, N-try) as independent variable with anthropometric, metabolic and hormonal parameters (i.e.BMI, glucose, insulin, HOMA-IR, total and HDl-cholesterol, triglycerides, basal GH, peak GH response and GH levels at 90 min after GHRH+arginine, fT3, fT4, TSH, IGF-1) as dependent variables.

## Results

**Fig 1:**
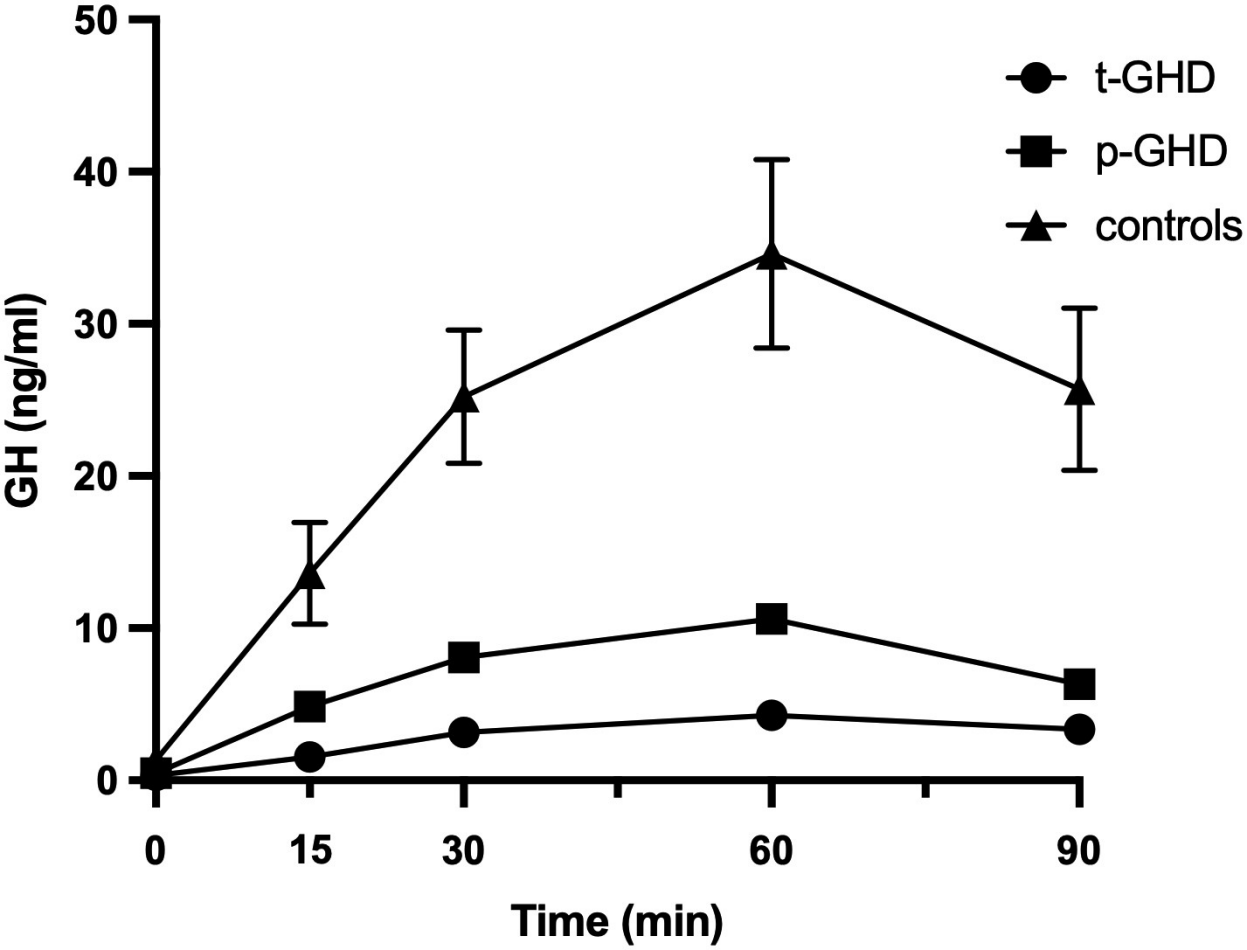
Average of the results of the GHRH/ARG test response in subjects enrolled in the study. Patients with t-GHD have a peak of less than 9.0 μg/L, those with p-GHD a peak lower than 16.0 μg/L and the controls have a response greater than 16.0 μg/L. Abbreviations: GHRH/ARG test: growth hormone-releasing hormone/arginine test; t-GHD: total GHD; p-GHD: partial GHD; ctrl: control subjects.

The GHRH/ARG combined test response in subjects enrolled in this study are depicted in Fig 1 as the average of the results. Mean ± standard error of the mean (SEM) age of the three groups were 50.28±2.22 years (total GHD group), 49.25±2.48 years (partial GHD group), 43.93±2.79 years (control group). Mean ± SEM BMI of the three groups were 31.05±2.47 kg/m^2^ (total GHD), 27.2±1.4 kg/m^2^ (partial GHD), 25.5±1.1 kg/m^2^ (controls).

Table 1 shows mean and SEM of metabolic parameters in patients with total, partial GH deficiency and controls. As metabolic parameters are concerned, a significantly increased HOMA-index was observed both in total (3.4±0.47) and partial (2.65±0.33) adult GHD in comparison to controls (1.51±0.19), p<0.05 (T test for unpaired data). A trend to worse lipid pattern was shown in total GHD.

**Table 1:** Metabolic parameters.

	TOTAL GHD		PARTIAL GHD		CONTROLS	
	*Mean*	*SEM*	*Mean*	*SEM*	*Mean*	*SEM*
**Glucose** (mg/dl)	84.92	3.07	83.81	1.98	80.50	1.74
**Insulin** (μUI/ml)	15.86*	2.06	12.65*	1.53	7.49	0.89
**HOMA-IR**	3.40*	0.47	2.65*	0.33	1.51	0.19
**Cholesterol total** (mg/dl)	189.56	5.43	194.25	6.66	195.00	7.25
**Cholesterol HDL** (mg/dl)	53.76	3.52	58.15	3.24	60.23	2.56
**Cholesterol LDL** (mg/dl)	122.75	8.90	113.20	7.97	112.31	2.56
**Triglycerides** (mg/dl)	110.64	8.25	96.70	7.68	88.73	7.69
**Uric acid** (mg/dl)	6.09*°	0.26	5.13	0.34	4.44	0.19

Mean and SEM of metabolic parameters in subjects enrolled in this study: total GHD, partial GHD and controls.

*p<0.05, total and partial GHD vs controls, using Student T test for unpaired data.

°p<0.05, total GHD vs partial GHD, using Student T test for unpaired data.

Table 2 shows mean and SEM of hormonal parameters. IGF-1 levels did not differ among the three groups (111.4±6.8 in total GHD, 127.4±7.63 in partial GHD, 122.77±9.49 ng/ml in controls) p<0.05 (T test for unpaired data).

**Table 2:** Hormonal parameters.

	TOTAL GHD		PARTIAL GHD		CONTROLS	
	*Mean*	*SEM*	*Mean*	*SEM*	*Mean*	*SEM*
**IGF-1** (ng/ml)	111.44*°	6.80	127.41	7.63	122.77	9.49
**fT3** (pg/ml)	3.02	0.08	3.18	0.07	3.04	0.09
**fT4** (pg/ml)	11.19	0.45	11.43	0.28	11.14	0.29
**TSH** (μUI/ml)	1.13	0.09	1.64	0.17	1.51	0.28
**ACTH** (pg/ml)	20.16	1.82	22.16	3.31	22.81	2.79
**Cortisol** (ng/ml)	91.22	9.94	116.55	11.45	109.57	10.80

Mean and SEM of hormonal parameters in subjects enrolled in this study: total GHD, partial GHD and controls.

*p<0.05, total and partial GHD vs controls, using Student T test for unpaired data.

°p<0.05, total GHD vs partial GHD, using Student T test for unpaired data.

**Fig 2:**
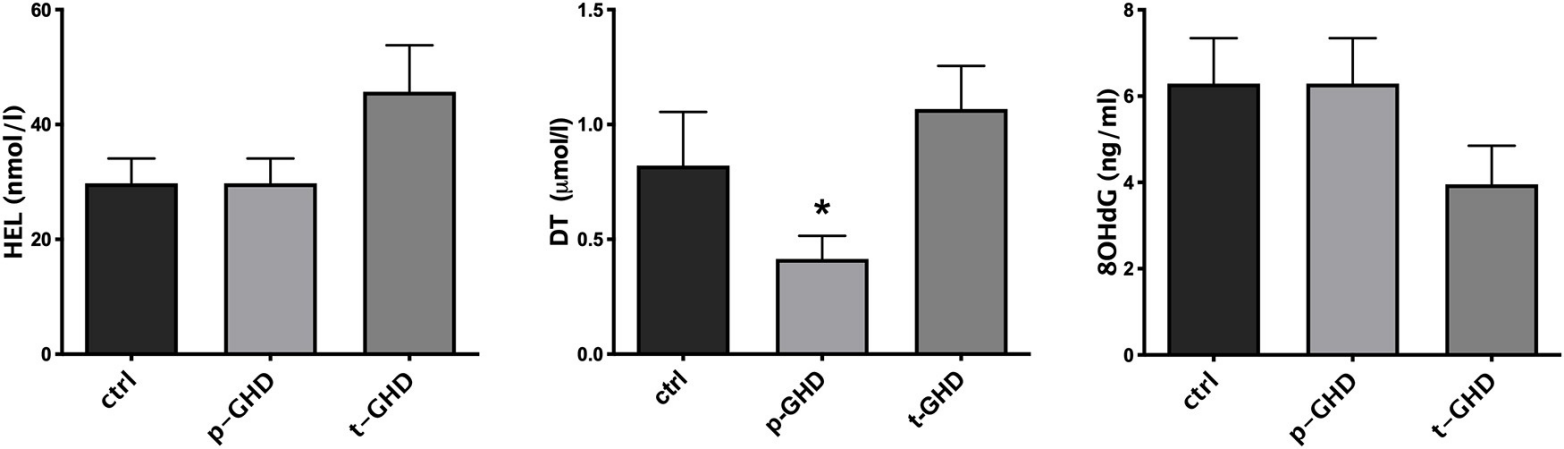
Mean ± SEM of the values of urinary Hexanoil-lysine (HEL), Dityrosine (DT) and 8-hydroxy-2 deoxyguanosine (8OHdG) evaluated in the three groups. *p<0.05 vs other two groups. Abbreviations: t-GHD: total GHD; p-GHD: partial GHD; ctrl: control subjects.

**Fig 3:**
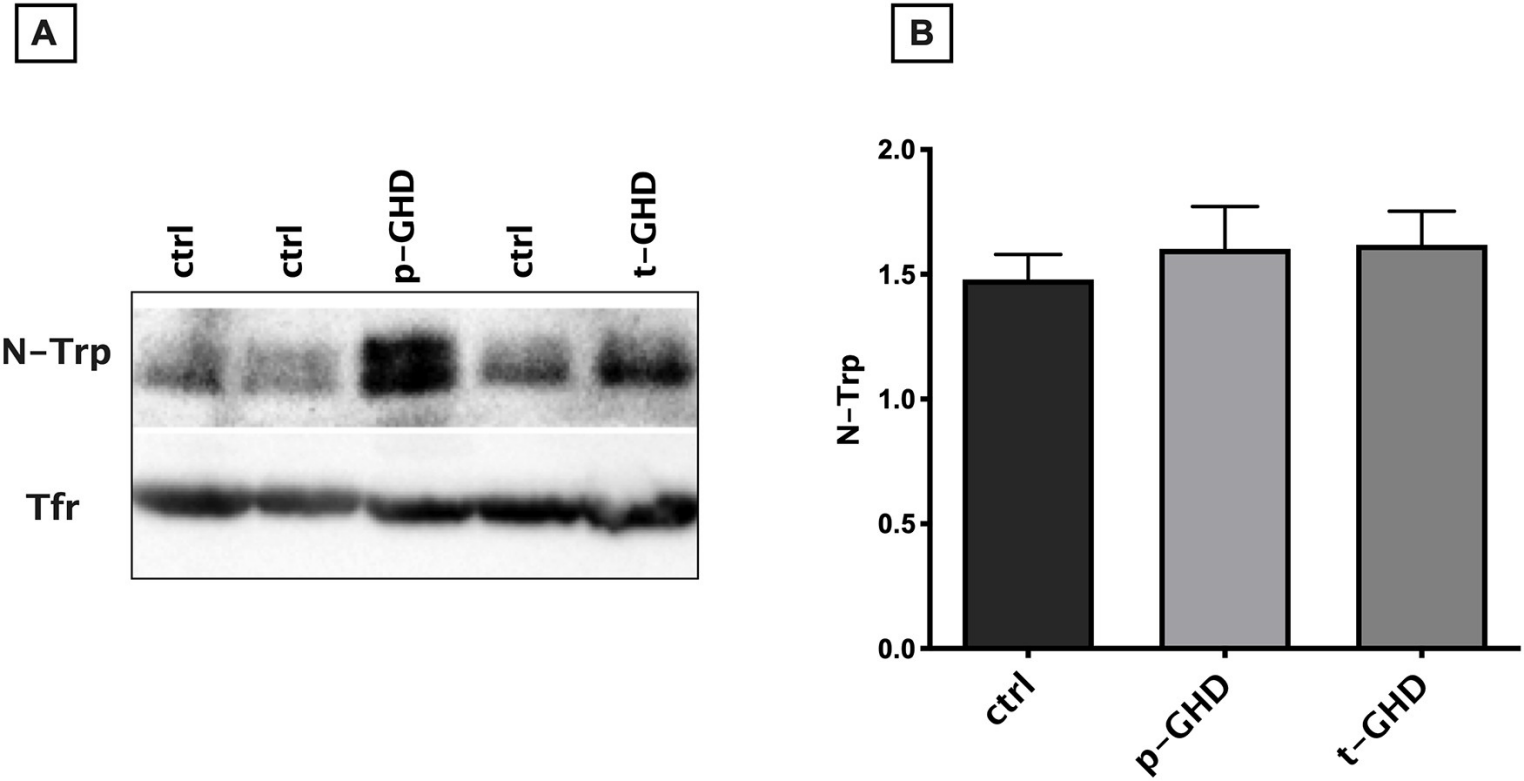
Western Blot and densitometric analysis of serum nitrotryptofan (NT) and transferrin (Tfr) levels in the three groups. (A) Western blot analysis. Blots were acquired with a *Chemidoc XPS* and the images were analyzed with the *ImageLab* software. Transferrin was used as an internal and normalization control. (B) Densitometric analysis from all the data analyzed. The relative densitometric expression of each patient was normalized with transferrin expression. Data are expressed as fold/increase (mean±SEM). Abbreviations: t-GHD: total GHD; p-GHD: partial GHD; ctrl: control subjects.

**Fig 4:**
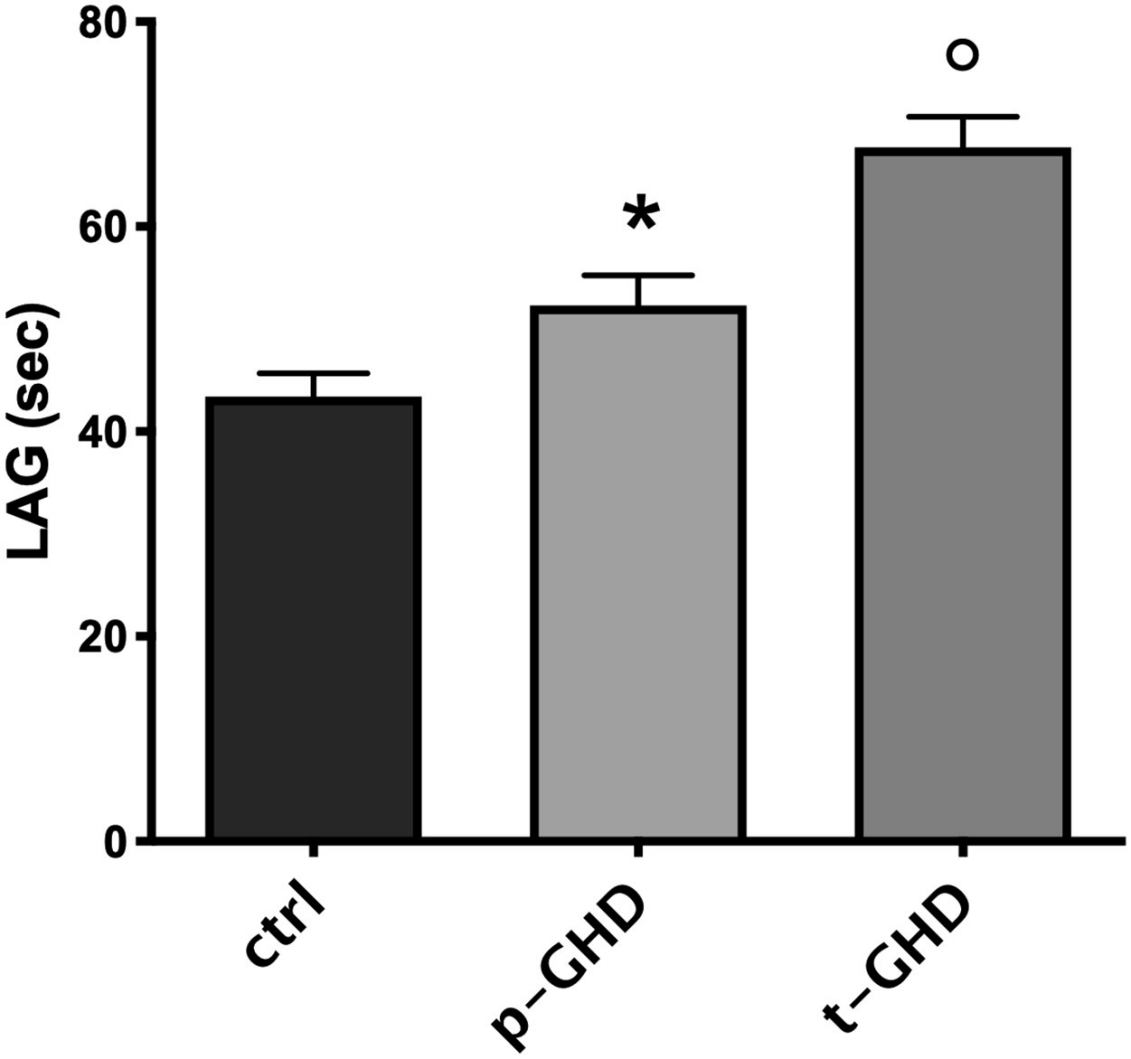
Mean±SEM of total antioxidant capacity, expressed by LAG (sec) in the three groups. *p<0.05 vs controls; °p<0.05 vs p-GHD and controls. Abbreviations: t-GHD: total GHD; p-GHD: partial GHD; ctrl: control subjects.

In the three groups the indexes of oxidative stress were also singularly compared in urine and serum of the subjects for each group. Fig 2 shows mean ± SEM of the values of urinary parameters (i.e. HEL, DT, 8-OHdG) in the three groups of patients. In evaluation of urine products of oxidation, a trend to increase in HEL in total GHD vs partial and controls was observed, although not significant. Values of 8-OHdG did not significantly differ among the three groups. On the contrary, significantly lower levels of DT in partial GHD vs total and controls were found (p<0.05- T test for unpaired data). A significant difference in N-Try was not found (Fig 3), while significantly greater values of TAC (p<0.05- T test for unpaired data) were observed in partial GHD vs controls, with a further significant increase in total GHD vs partial GHD (Fig 4).

Interestingly, data occurred in correlation analysis, depicting different patterns in control and partial GHD, on one side, and total GHD on the other one. For seek of clarity, in controls LAG values showed a positive correlation with BMI and insulin (r = 0.49 p = 0.03 and r = 0.51 p = 0.02, respectively) and an inverse correlation with GH stimulated levels (peak and 90 min) (p = -0.47 p = 0.02 and r = -0.44 p = 0.02, respectively); in partial GHD, LAG positively correlated with BMI and HOMA-IR (r = 0.53 p = 0.03 and r = 0.65 p = 0.008) and an inverse correlation with GH levels (90 min) (r = -0.43 p = 0.03). Finally, a negative correlation was found between HEL and GH at 90 min both in controls (r = -0.54 p = 0.006) and partial GHD (r = -0.60 p = 0.002), and between N-try and GH at 90 min in (r = -0.52 p = 0.03).

These correlations were not present in total GHD patients. On the contrary, an inverse correlation was observed between HEL and DT with HOMA-IR (r –0.42 p = 0.04 and r –0.42 p = 0.04, respectively), and a positive correlation between HEL and basal and peak GH (r = 0.79 p = 0.0004 and r = 0.43 p = 0.04, respectively), between 8-OH-dG with basal GH (r = 0.56 p = 0.03), and between N-Try and BMI (r = 0.59 p = 0.02).

## Discussion

To the best knowledge of the authors, this is the first report that shows parameters of macromolecular oxidative damage in adult GHD subjects. In serum, we evaluated N-Try, as index of protein oxidative damage; in urine samples, we evaluated HEL, DT and 8-OHdG, as parameters of oxidative damage of lipid, proteins and DNA, respectively. There were no significant changes among the three groups, except for DT that, unexpectedly, was significantly lower in partial GHD versus the other two groups (U-shaped pattern). This datum could be explained considering the antioxidant systemic response of the body, which was assessed in our study by TAC (expressed as Lag phase in sec). Indeed, already in partial GHD, TAC is significantly greater than controls, yet antioxidants are still able to compensate oxidative damage (as indicated by lower DT urinary levels). Oxidative stress is further increased in total GHD which presented the highest TAC levels (as a result of an increased production of antioxidants to balance the enhanced OS), a trend in increased HEL and increased urinary DT production (vs partial GHD), suggesting a partial compensation despite a remarkable antioxidants production.

Additionally, few studies pinpointed on the relationship between OS and adult GHD. Pre-pubertal GHD showed a significant reduced TAC and vitamin E correlated to the reduced levels of IGF-1/ IGF-BP3 while malondialdehyde (MDA), another marker of oxidative stress, was significantly increased and inversely correlated to IGF-1. The re-evaluation of these parameters after 1 year of recombinant GH (rGH) therapy showed an increase of TAC and a decrease of MDA, both returned to normal levels [9]. Higher peroxide levels and lower TAC was found in GHD patients by Scacchi et al, however no direct correlation with IGF-1 was highlighted. After 4 months of follow up with rhGH treatment, both peroxide levels and TAC were restored to control values [10]. On the contrary, the group of Gonzàlez-Duarte found higher TAC levels in adult GHD patients than controls [32]. The discrepancy between these results and those reported in the present study is probably due to different TAC evaluation method and the different stages in which the disease was assessed.

Therefore, considering the complex picture and putting all data together, we can suggest that also partial GHD exhibits antioxidant systems reactivity, suggesting a condition of oxidative stress. We cannot establish if partial and/or total GHD represent different stages of the natural history of the syndrome (but longitudinal studies can clarify this point).

Other plasmatic protein damage indexes, such as nitro-tyrosine and carbonyls, have already been considered in other study, even though no difference was detected between adult GHD and healthy controls. Thus, data on protein serum damage indexes, represented here by N-Try, are congruent with that reported by Ozbey [33]. Furthermore, the same study report a significant increase in oxidized LDL, in turn associated to a worse atherosclerotic picture compared to healthy controls, in adult GHD, a result afterwards confirmed by several studies [10,32,33]. In striking contrast, Smith et al. reported in adult GHD reduced lipid peroxidation, evaluated both as plasma lipid hydroperoxides and LDL susceptibility to peroxidation [34]. Despite, in this study lipid damage was not assessed by plasmatic indexes of lipid peroxidation, urinary HEL have been analysed, showing a trend in increased production, yet not significant, in total GHD group. A larger study population may support a better identification of this phenomenon. Importantly, Ozbey et al. report that oxidized LDL levels in adult GHD patients match with the duration of the disease in a group of patients with a median duration from the diagnosis of hypopituitarism of 96 months. This aspect could be decisive in the interpretation of data. Indeed, Gonzàlez-Duarte et al. pointed out that OS may be present since the early stage of the disease and may be influenced by time-course of the disease. The same study confirmed the increase in oxidized lipoproteins production, highlighted the consumption of the antioxidant glutathione and the reduced/oxidized glutathione ratio and indicated, as previously reported [35], a worse endothelial function in adult GHD [32].

Surprisingly, none of already cited paper has analyzed partial GHD condition, which, according to our results, seems to reveal an initial state of oxidative stress, although still compensated. Metabolic parameters, either, present some alterations both in total and in partial adult GHD, thus confirming by Colao et al. observations [20–22].

Correlation studies highlighted a differential pattern of association between macromolecular damage and hormonal/metabolic parameters in the three groups studied, despite an unequivocal scenario was not already clear, very likely for the small number of subjects involved and therefore the difficulty to establish a direct cause-effect relation. While in controls and partial GHD subjects, OS seemed to be related to BMI and insulin-resistance and inversely related to GH levels, the correlations were different in total GHD, in which other mechanisms seems to be involved. The correlation with GH levels is difficult to elucidate, but we underline that the patients studied showed very low levels of the hormone. Moreover, evidence for such different patterns has been previously described [13].

The strength of this study lies in the novelty of the topic and in the parameters evaluated in such subjects, extending our previous reports on oxidative status in GHD [13]. Thus, we can suggest that oxidative stress is increased both in partial and total GHD, hypothesizing that lack of compensation by antioxidants can be associated to the recognized complications of this syndrome.

In spite of all precautions, our study may be still subject to certain biases, and some main potential restrictions should be considered. The number of subjects in the three groups is slightly small, so the statistical power of the study is limited; consequently, our findings will need to be confirmed in a larger population and will help to delineate the impact of OS in GHD condition. Therefore, the study design and the power analysis cannot draw a cause-effect relationship. In the present study, the only assessment of antioxidants is TAC, which is an expression of non-enzymatic antioxidants, even though enzymatic antioxidants (e.g CAT, GSHPx, and SOD) may be relevant in human being. The moment of the disease history in which the patient is evaluated may affect antioxidants production and macromolecular damage. Finally, more data and longitudinal studies are still needed in order to better characterize partial GHD and its possible evolution to total GHD. Anyhow, the relationships between OS and adult GHD merit further studies to develop targeted personalized therapy, including medical interventions with long-term hormonal replacement with GH.

## Supporting information

S1 File Blot results. Original unadjusted images underlying blot made for N-Try serum evaluation.
(PDF)
