# Supplementary figures and images for: Evaluation of oxidative stress effects on different macromolecules in adult growth hormone deficiency
Source: PLoS One. 2020 Jul 20;15(7):e0236357. doi: 10.1371/journal.pone.0236357 (PMC7371168; doi:10.1371/journal.pone.0236357)

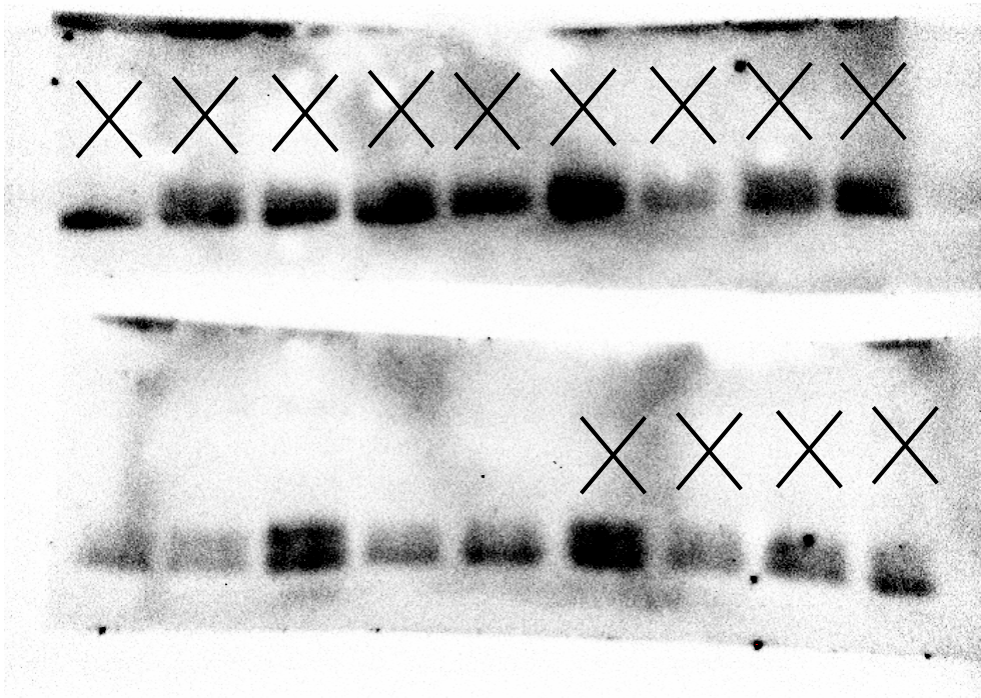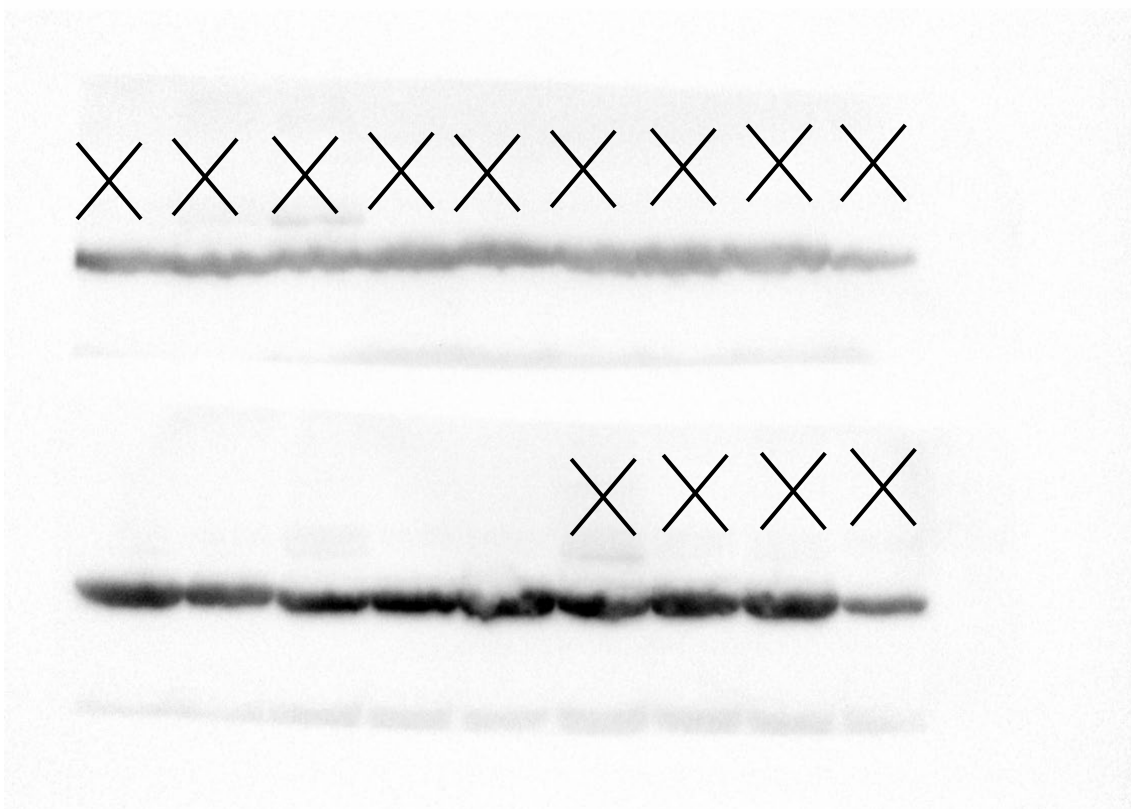

Supplement: S1 File — Original unadjusted images underlying blot made for N-Try serum evaluation. (PDF) [file pone.0236357.s001.pdf]
